# Running-Related Injury From an Engineering, Medical and Sport Science Perspective

**DOI:** 10.3389/fbioe.2020.533391

**Published:** 2020-09-30

**Authors:** Maria Papagiannaki, Efthimios Samoladas, Stergios Maropoulos, Fotini Arabatzi

**Affiliations:** ^1^Department of Physical Education and Sport Science, Serres, Aristotle University of Thessaloniki, Thessalonik, Greece; ^2^Department of Orthopaedics, Medical School, Aristotle University of Thessaloniki, Thessaloniki, Greece; ^3^Department of Mechanical Engineering, University of Western Macedonia, Kozani, Greece

**Keywords:** footwear selection, impact cushioning, trauma precursors, cytokines, running biomechanics

## Abstract

Etiologic factors associated to running injuries are reviewed, with an emphasis on the transient shock waves experienced during foot strike. In these terms, impact mechanics are analyzed from both, a biomechanical and medical standpoint and evaluated with respect injury etiology, precursors and morbidity. The complex interaction of runner specific characteristics on the employed footwear system are examined, providing insight into footwear selection that could act as a preventive measure against non-acute trauma incidence. In conclusion, and despite the vast literature on running-related injury-risks, only few records could be identified to consider the effect of shoe cushioning and anthropometric data on injury prevalence. Based on this literature, we would stress the importance of such considerations in future studies aspiring to provide insight into running related injury etiology and prevention.

## Introduction

The purpose of this review is to examine the current literature on running related injuries and stipulate whether these can be mitigated through proper footwear selection. As current literature is naturally dominated by the sport science perspective, we draw on two complementary point of views, engineering and medical, to provide insight into the underlying impact mechanics and injury precursors, that may catalyze a better understanding of injury risk and prevention (Hreljac, 2004).

1216 articles, as to overlapping information (duplicates) or a lack of relevant scope. 31 Review papers and 61 research articles were considered as eligible, by two of the co-authors, independently, coming to a consensus as to their inclusion, based on the predefined selection criteria. Additionally, two ASTM standards relevant to the purpose of the study were included.

Based on the data collected, the basic principles of impact mechanics are explored, introducing an engineering point of view on the stressors developing during running. These, taken into consideration with running biomechanics and kinematic patterns, facilitate the interpretation of the loads imposed on a runner, as well as the identification of how these, causes or aggravate potential symptoms.

Following this, the etiologic factors associated to running injuries are reviewed, while context on injury precursors and morbidity is provided from a medical perspective. There is no literature, correlating these two aspects with footwear selection (Hannigan and Pollard, 2020), which is attempted in the concluding chapter of this review.

**FIGURE 1:**
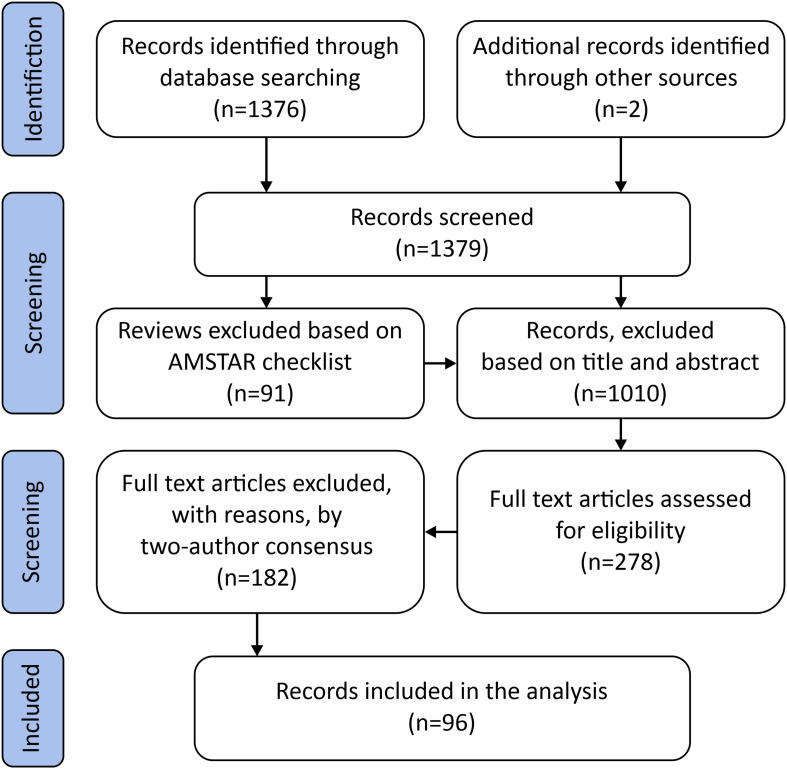
PRISMA flow diagram.

An overview of the literature review flow chart is presented in Figure 1.

For the purpose of this review 1376 articles containing the keywords “running injury,” “running biomechanics,” “impact mechanics,” “impact cushioning,” and “footwear selection” were randomly retrieved from PubMed and Scopus to provide context for this review. 160 of the articles were review articles and assessed with the AMSTAR checklist. AMSTAR compliant reviews were evaluated along with the remaining.

## Impact Mechanics and Their Correlation to Running Biomechanics

### Impact Force Measurement and Sissipation

Intended as shock-mitigating materials, midsole systems are expected to absorb kinetic energy and do so, like any cushioning structure (Michailidis et al., 2014), in a non-linear manner. The initial approach to force measurement and the subsequent evaluation of impact attenuation of technical footwear systems, employed force platform measurements (Lloyd and Wu, 2013). Sub-sole experimentation, however, was swiftly discarded as methodologically “flawed,” due to limitations associated to the evaluation of the foot/ground interface and the absorbed energy allocation (McMillan and Payne, 2008). Succeeded by the use of accelerometers, mounted on individual test subjects (Gruber et al., 2014) and later on by “on-sole” testing techniques (Tsouknidas et al., 2017), recent literature is now in agreement with latest international testing standards (ASTMF 1614 – 99, 2006; ASTMF 1976 – 06, 2006).

### Impact Mechanics

During running, our lower extremities are, like any object colliding with a rigid/semi-rigid surface, exposed to transient forces that significantly exceed our weight. The principles behind this, exhibit characteristics typical to low-speed semi-rigid-body impacts, with the resultant forces acting in the opposite directions of the foot colliding to the ground. According to Newton’s second law, the force (*F*) acting on a foot during running, should be proportional to, and in the direction of its momentum change rate (*dMV*), with respect to time (*t*).

(1) $$F_{\left(t\right)}=\frac{d\,\left(M\,V\right)}{d\,t},$$

where *M* represents the decelerating mass [corresponding to ≈3.6 kg in a typical adult subject (Ker et al., 1989)] and *V* the impact velocity. It should be noted that, in this rather simple consideration, the impact depends on the incident relative velocity and the runner’s mass.

To analyze the occurring change in momentum, the conventional approach in mechanics would be to consider deformations as negligible and small, which greatly simplifies the analysis, as the change in velocity can be calculated without integrating accelerations over the contact period (Strong, 2018).

In the presence, however, of a cushioning system, such as padded midsole, the impact attains some characteristics of high velocity impacts i.e., manifesting as strains of the visco-elastic material (Wingate et al., 1993), which requires the consideration of inertia, whereas gait kinematics must also satisfy equations of motion. Finally, the propulsive nature of the impact, restricts the use of the kinematic coefficient of restitution, as frictional forces within the shoe-ground interface oppose the initial slip (Stewart, 2010). As a consequence of this complexity, the impact mechanics of footwear are conventionally approached with notable simplifications.

Existing literature largely bases experimental findings on the “law of conservation,” which states that energy cannot be destroyed, merely transferred among bodies or changed into another form (Baroud et al., 1999). Limited to time-independent assumptions, footwear research conventionally evaluates results in terms of total or max values of energy, force and deformation (Zhang et al., 2005).

This essentially means that only limited information as to the force mitigating properties and/or the energy retention of footwear can be elucidated through conventional experimentation and thus, new paradigms have to be sought. In this context, Finite Element (FE) models have been long introduced as cohesive elements in the interpretation of experimental investigations in biomechanical systems, providing insight into complex phenomena, ranging from the macro level e.g., spine biomechanics (Tsouknidas, 2015) and masticatory system (Michailidis et al., 2013), to the cellular one (Lim et al., 2006). Recent FE studies aspired to interpret the underlying mechanisms, based on which mechanical forces are attenuated during impact, pointing out the importance of considering shoe design with respect to strike-pattern (Drougkas et al., 2018).

### Footwear Related Running Biomechanics

Since impact mechanics (during running) are a multifactorial phenomenon, the interpretation of the force allocation to the involved structures (footwear, musculoskeletal system, etc.), also warrant an understanding of the underlying kinematics.

Despite the term running biomechanics referring to the kinematics of the entire human body during running, thus also entailing e.g., arm and trunk posture, footwear biomechanics can be restricted to the movements of the lower extremities. This periodic kinetic chain, following the initial impact of a foot with the ground until it reconnects with the surface at the end of a cycle, is called gait (Dicharry, 2010). Impact mechanics naturally focus on the stance phase of this cycle, it should be noted though, that the airborne swing and float phases strongly influence the impact that follows as well, as multiple factors like stride length, have been associated to cadence and velocity (Schubert et al., 2014), an increase of which will result in a more forceful impact (Dugan and Bhat, 2005).

Cushioning systems of technical running shoes are conventionally designed around these concepts (Tsouknidas et al., 2017), while considering the narrow base width support during the occurring impact (Nicola and Jewison, 2012). This results in a variety of footwear systems, as runners are classified by three different strike-patterns (Almeida et al., 2015), denoting the support area during impact: (a) heel-strike, where initial contact is made through the calcaneus, (b) midfoot strike, engaging the posterior and anterior portions of the foot simultaneously, and (c) forefoot-strike, during which runners primarily land on their metatarsals. Pronation in also of importance, as it essentially describes the motion of the lower limbs in the sagittal, frontal and transverse planes (Hintermann and Nigg, 1998) and abnormal motion patterns (e.g., overpronation) have been associated with trauma in the lower extremities (Lysholm and Wilander, 1987). Both combined, strike pattern and pronation, essentially dictate joint stabilization and intrinsic shock absorption and thus are vital considerations in the evaluation of impact allocation between footwear and the runner’s limbs.

Despite contact not being invariably made by the heel, nor the foot always rolling inward at about 15 percent (normal pronation), there are some activities, that favor specific gait patterns. Studies, for instance, have shown that 88,9% of all runners are biased toward heal-strike when engaging long distances, irrespective to what their foot-strike would be over shorter ones (Larson et al., 2011). This is a vital consideration for injury risk and morbidity studies, as long-distance runners provide a platform for the evaluation not only of injury etiology, but also propagation. This aspect is not always considered in controlled trials (Withnall et al., 2006; Theisen et al., 2014). Footwear designed for this type of activity, accommodates midsole systems capable of attenuating the strenuous overloading that is expected to occur during longer distances, whilst compensating for weight and landing stability.

Different footwear systems, have also been associated to energy storage and retention as well as energetic cost of running (Hoogkamer et al., 2018). Regardless of the methodological approach of the study e.g., the focus on running velocities that significantly exceed those of recreational athletes, their findings are a clear indicator that running biomechanics and physiology are affected by a change in compliance and resilience of the employed footwear system.

As a result, footwear is often tuned to specific runner characteristics, as studies have shown that they are optimized both for a specific gait type [e.g., heal strike (Tsouknidas et al., 2019)] as well as narrow body weight ranges (Tsouknidas et al., 2017). Experts across disciplines from biomechanics to medicine (Lieberman et al., 2010; Almeida et al., 2015), agree that running mechanics are altered in myriad more ways, depending among others on running speed, terrain and anthropometric data (height and weight), to joint stiffness and cushioning system employed (Nigg et al., 2003).

In a nutshell, our nervous system analyses sensory feedback, associated to transient shock waves that are dissipated through our musculoskeletal system in a highly adaptive manner, and recruits muscles as needed, to alter kinematics. This shock moderating behavior, is a subconscious attempt to find the path of “least resistance,” thus mitigating the impact sensed as a potential injury risk (Robbins and Gouw, 1990). This may manifest in different landing patterns, a change in cadence or vertical oscillation, ground contact time, or in asymmetric upper body movements (one arm swinging out farther than the other) to compensate for momentum irregularities. Nevertheless, how these modulations affect injury risk, remains unclear.

## Running Related Injuries

Arguably one of the most popular athletic activities worldwide (Running USA, 2014), running was bound to elicit interest concerning the incidence and epidemiology of running-related injuries (Fields et al., 1990; Gerlach et al., 2008). Despite the fact that experience can lessen the risk of prevalence (Saragiotto et al., 2014), the transient impulses generated during running, may rapidly turn any potential health benefits into trauma. As a result, depending on intensity and duration, exposure to repetitive impacts has been documented to trigger a variety of injuries, ranging from muscle tears and stress fractures to degenerative joint disease (Folman et al., 1986; Whittle, 1999). As running is adopted by millions worldwide, replacing physical inactivity, injury prevention becomes more and more prevalent as the involved risk cancels-out any potential health benefits.

### Epidemiology and Statistics

Incidence of trauma has been recorded to manifest approximately 17.8 times for every 1000 h of training (Videbæk et al., 2015), on a weighted average across different types of runners (e.g., novice/recreational or elite/sub-elite athletes). It should be noted though, that distance, seems to be of significant relevance in injury incidence. Long distance runners exhibited a lower prelevance of 2.5 – 7.2, which was not only recorded in ultra-marathon runners (Krabak et al., 2011), but also in long-distance track and field athletes (Lysholm and Wilander, 1987). Sprinters, middle-distance runners displayed a different trend, with injuries appearing as often as 26.3 times (Bennell et al., 1996).

There are, however, some inconsistencies in literature on running related injuries per 1000 h of running. This might be due to an accumulation of experience (Videbæk et al., 2015), as novice runners may evolve toward the end of the study into recreational/more experienced ones. The argument in favor of this stipulation, is that injury incidence is far more homogeneous in recreational runners and thus seems to be unbiased by the study’s follow-up duration (Wen et al., 1998). Based on this alone, it stands to reason that proper training could significantly reduce injury prevalence, a notion which has been confirmed by literature (Hreljac, 2005).

Injury as a term in-itself, however, is another aspect in which literature seems to lack consensus. Most studies classify compulsory time-loss from training as an injury, which is, however, a subjective criterion, spanning from a day (Buist et al., 2010) to 1 week (Larson et al., 2011). With the etymology of injury portraying physiological damage that interferes with one’s ability to run (Valliant, 1981), retrospective uncontrolled studies would provide a subject specific alternative, being arguably more tangible. Adversely to such epidemiologic studies, which are far more difficult to conduct and subject to recall bias (Reinking et al., 2007), considering the actual need for medical attention as injury, is only applicable whilst monitoring ultra-marathons (Wen et al., 1997) and thus, not an ideal criterion. Lastly, physical pain does not qualify as injury on its own (Whittle, 1999), but has been considered the injury definition by several studies (Bovens et al., 1989; Bennell et al., 1996; Wen et al., 1998).

Injury trends are commonly associated to joint overloading with the knee being the anatomical site of interest in more than 40% of trauma cases (Messier and Pittala, 1988), with an equivalent incidence to the ankle, foot and lower leg combined (Messier and Pittala, 1988; Williams et al., 2008). The remaining 20% has been reported to occur above the knee. Notably most injuries are tied to running style (Goss and Gross, 2012) thus indicating that adjusting one’s gait to the type of running (short vs long distance), could hold the potential of reducing injury prevalence. This is also supported by the type of injury, as acute trauma (e.g., fractures and ankle sprains) is less frequent than overuse injuries of the musculoskeletal system (Stanish, 1984), e.g., Achilles tendinitis, patellofemoral pain syndrome, plantar fasciitis and medial tibial stress (shin splints).

### Etiology

While the anthropometric characteristics of individual runners vary significantly, as does their gait, anatomical factors have been refuted as risk factors, as a clear correlation of these variables to injury prevalence could not be established (James, 1998). Although runner anatomy is likely to alter the impact mechanics during running, there are some studies indicating that these could be compensated by proper footwear selection (Tsouknidas et al., 2017). Biomechanical factors have been reported to have a more direct correlation to running injuries, e.g., excessive pronation has been indicated as a contributing factor to overuse running injuries in multiple clinical studies (Jones, 1998; Ferber et al., 2009).

There is, however, a consensus in literature that training errors associated to stress-frequency phenomena (i.e., fatigue loading), are likely the primarily etiology of running related injury (James et al., 1978; Paty, 1994). It should be noted, that despite this affinity, lessening the repetitive forces that are propagated through the musculoskeletal system during running, in itself, would not necessarily avoid injury. The key to understanding trauma morbidity is associated to the structure’s injury threshold, a concept defined by Wolf’s principle (Wolff, 1892). According to this, any anatomy subject to stress, is bound to remodel as to withstand future loading in a more efficient manner, given that the applied stress lies within the strength limit of the tissue and an adequate time period is provided to set this remodeling forth. As a result, overloading may occur if either one of these aspects (time or load magnitude) disturb the equilibrium (Hreljac, 2005). Trauma etiology and propagation, however, also differs significantly among tissue types. In this context, soft tissues (e.g., tendons) for instance, are susceptible to intrinsic loads (Wright et al., 1998), that have been measured between 6.1–8.2 times the subject’s body weight (BW) (Scott and Winter, 1989). It has been argued that alterations in muscle recruitment, due to footwear modification, could very well affect soft tissue injury prevalence (Azevedo et al., 2009), while a recent study has pointed out that midsole stiffness could potentially affect soft tissue mechanics during long-distance running performance (Cigoja et al., 2020). Bone injuries on the other hand, acute or chronic in nature, entail higher loads ranging up to 14.1× BW (Scott and Winter, 1989).

A recent review (Nielsen et al., 2012), has identified training related errors such as: as intensity, excessive running duration or too steep increase rates in training duration/distance as injury risk factors. This would be in agreement with the aforementioned impact and running bio-mechanics and as such, also interrelated with the terrain and footwear compliance.

Several other variables have been hypothesized as injury risk factors, e.g., arch height, mis-alignment of lower extremities, ankle range of motion, ankle flexibility etc. but literature on these topics remains controversial (Warren and Jones, 1987; Cowan et al., 1989; Wen et al., 1997; Hreljac, 2005), despite the fact that runners exhibiting multiple of these anthropometric characteristics might be more susceptible to injury. Similarly, poor low back and posterior thigh flexibility have been suggested as etiologic factors in running injuries (Brody, 1980; McKenzie et al., 1985), but these suggestions were rejected by later studies (Messier and Pittala, 1988).

Pre-existing trauma is of course highly prevalent, as even a history of exercise-related pain increases the likelihood of relapse (Bennett et al., 2012). Strong evidence also exists, that runners with a history of previous injury are at higher risk of follow-up trauma than athletes with no pre-existing medical indicators (Van Middelkoop et al., 2008).

### Precursors and Morbidity

From a biomechanical standpoint, muscle and joint overloading is bound to induce physiological changes of the underlying tissue (Bader et al., 2011). Common to long-distance running, such stressors can elicit inflammatory responses which spam from muscle soreness, to cell apoptosis followed by collagen degeneration (Loening et al., 2000) and chronic trauma.

Despite the fact that any type of training may be accompanied by localized inflammation, which is regarded as a protective response to onset tissue damage (Hung and Suzuki, 2017), strenuous exercise can result in cytokine release into the circulation, which induce a pattern of immunological/pathogenic responses similar to sepsis (Pedersen, 2000). Increased proinflammatory cytokine levels, leukocyte infiltration and oxidative stress are well-known exercise-induced inflammation precursors (Suzuki, 2017b). Tumour necrosis factor (TNF)-a, is the first cytokine to peak, following considerable tissue damage (Suzuki, 2017a) followed by the systematic release of interleukin (IL)-1 and IL-6 (Pedersen and Febbraio, 2008).

Natoli et al. (2008) suggested that injury at the knee joint could occur during running as a delayed biological response to stress (Duda et al., 2001) and loading rate (Aspden et al., 2002) values, that would otherwise be below the injury thresholds for this type of tissue. This indicates that cartilaginous tissue exposed to seemingly non-dangerous loading conditions could be indeed prone to overloading injuries duration long-distance running, if not mitigated appropriately.

This theoretical background could be used to determine appropriate biomarkers to evaluate the effect of exhaustive endurance exercise. Due to the cytokine released sequence, TNF-a would be suitable to indicate pro-inflammatory responses, both early-on as well as in terms of morbidity. IL-1 could be used complementary, to distinguish acute trauma (e.g., strains) form other running related injuries. This is due to the fact that interleukin release is not related to exercise intensity but duration (Suzuki et al., 2002; Pedersen and Febbraio, 2008) and thus, these cytokines could be considered as a biomarker for fatigue-induced or systemic overloading injury. However, IL-6 directly inhibits the expression of TNF-a and IL-1 and thus the upregulation of major inflammatory mediators (Pedersen, 2000). As a result, IL-6 levels should also be monitored as they prevent signal transduction of the pro-inflammatory cytokines (Barton, 1997), whilst considering that IL-6 production during exercise is significantly higher than any other cytokine.

Cytokines can be identified and measured in plasma collected after exercise (Sprenger et al., 1992) and thus their use as injury precursors mainly depends on establishing their diagnostic cut-offs (Monastero and Pentyala, 2017). Since cytokine profiles and kinetics are subject-specific, determining their normal levels is vital to provide systematic insight on how they modulate biochemical pathways to running related injury and its morbidity.

## Athletic Footwear

A proactive approach to injury prevention, could be both training related or of medical nature (frequent screening), but any of these must be paired with an accessible choice for most runners: proper footwear selection and fitting. Midsole cushioning technology has the potential to greatly alter reaction forces during running (Maropoulos et al., 2017), while other factors associated to shoe construction (e.g., shoe drop and midsole density) can affect the lever arm about the subtalar joint axes, which has been determined to affect running related injuries (Stacoff et al., 1988) and running in inappropriate footwear has indeed been associated to injury (Komi et al., 1987; Wilk et al., 2000).

Heidenfelder et al. (2009) argued that the shock attenuating capacity of technical footwear deteriorates consistently during the first 600 km of running, whereas pronation patterns adapt to such changes early on. Since footwear stiffness is widely accepted to affect running biomechanics (Hardin et al., 2004), it stands to reason that worn-down midsoles will do so as well, thus directly influencing injury prevalence among runners. A cross-evaluation of experimental and *in vivo* studies (Heidenfelder et al., 2009; Kong et al., 2014), would sustain the notion that running biomechanics are affected at higher mileages (more worn-down shoes), whereas cushioning properties tend to fade at a significantly higher rate, thus restricting the effectiveness of technical footwear systems to a shorter life span.

The expected life span of a running shoe is, however, a controversial topic among athletes, researchers and footwear manufacturers. Very little information has been documented on this subject, restricting the ultimate choice to personal preferences and experiences (Muendermann et al., 2002). Based on the fact that our body’s sensory mechanisms cannot perceive changes in midsole stiffness below 15 kN/m, which is significantly lower than the stiffness values of commercially available cushioning systems, it becomes obvious that reductions of the cushioning capacity of a shoe, are likely to go unnoticed even by elite runners!

As a result, worn down footwear system might still be identified as comfortable while exhibiting a significant rise of impact magnitude. This denotes another reason why uncontrolled studies (e.g., based on questionnaires), are ineffective in correlating footwear systems to injury prevalence.

From a medical standpoint, injury seems more likely to occur during strenuous running and thus, intensity and duration of running are a vital aspect of injury prevalence and morbidity. There are some studies hinting at this, by associating overuse to injury (Stanish, 1984), consequently, footwear selection becomes even more prevalent for long-distance runners.

Taking these considerations together with recent findings, that the metabolic cost of running decreases about 1% for 100gr weight loss per shoe (Franz et al., 2012) and how excess weight is bound to affect momentum change rates during impact, suggesting that the selection of lightweight, highly compliant, and resilient footwear for competitive distance runners.

Our perspective would be that despite the complex interaction of a plethora of factors that are prevalent to injury incidence, most non-acute trauma should be preventable through proper preparation, whether this is associated to footwear selection, or tuning one’s training to his experience and injury threshold. It should, however, be mentioned that the latter (stress-frequency relationship) is a multifaceted problem as different anatomical structures exhibit varying (subject-specific) threshold levels and are exposed to dynamic, multi-dimensional loads. Footwear selection should, nevertheless, be taken into consideration of future studies aspiring to provide insight into running related injury etiology and prevention. A randomized controlled trial, performed on 401 participants, showed that insoles of different cushioning capacity did not affect injury risk in a statistically significant way (Withnall et al., 2006). However, in line with similar studies (Theisen et al., 2014), no anthropometric characteristics (e.g., body mass) were considered, thus shoe allocation was not performed in a subject specific compliant way. This has been stressed to affect the shock mitigating capacity of athletic footwear (Tsouknidas et al., 2017) and should thus be considered in future studies. Notably, a recent study protocol (Malisoux et al., 2017) shows, that this limitation has not gone unnoticed by other groups as well.

It should be noted that the focus of this study is not performance- but injury-related. In this context we would suggest the selection of a more compliant midsole system for long distance runners within lower bodyweight ranges and footwear of medium compliance for mid-weight ranged runners. Despite the tremendous attention among athletic footwear manufacturers being lately directed toward energy return, we would recon that midsoles focusing on cushioning rather than turning impact into stride energy, would be preferable to avoid injury, despite requiring more physical effort to cover longer distances.

## Author Contributions

All authors contributed equally. MP provided the comprehensive literature review, which was screened and put in context by the complementary expertise of the remaining authors (Medical, Engineering and Sport Science).

## Conflict of Interest

The authors declare that the research was conducted in the absence of any commercial or financial relationships that could be construed as a potential conflict of interest.
